# Functional Vascular Changes of the Kidney during Pregnancy in Animals: A Systematic Review and Meta-Analysis

**DOI:** 10.1371/journal.pone.0112084

**Published:** 2014-11-11

**Authors:** Joris van Drongelen, Rob de Vries, Frederik K. Lotgering, Paul Smits, Marc E. A. Spaanderman

**Affiliations:** 1 Department of Obstetrics and Gynecology, Radboudumc, the Netherlands; 2 Systematic Review Centre for Laboratory animal Experimentation, Radboudumc, the Netherlands; 3 Department of Pharmacology and Toxicology, Radboudumc, the Netherlands; 4 Department of Obstetrics and Gynecology, Research School GROW, Maastricht University Medical Centre, Maastricht, the Netherlands; INSERM, France

## Abstract

Renal vascular responses to pregnancy have frequently been studied, by investigating renal vascular resistance (RVR), renal flow, glomerular filtration rate (GFR), and renal artery responses to stimuli. Nonetheless, several questions remain: 1. Which vasodilator pathways are activated and to what extent do they affect RVR, renal flow and GFR across species, strains and gestational ages, 2. Are these changes dependent on renal artery adaptation, 3. At which cellular level does pregnancy affect the involved pathways? In an attempt to answer the questions raised, we performed a systematic review and meta-analysis on animal data. We included 37 studies (116 responses). At mid-gestation, RVR and GFR change to a similar degree across species and strains, accompanied by variable change in renal flow. At least in rats, changes depend on NO activation. At late gestation, changes in RVR, renal flow and GFR vary between species and strains. In rats, these changes are effectuated by sympathetic stimulation. Overall, renal artery responsiveness to stimuli is unaffected by pregnancy, except for Sprague Dawley rats in which pregnancy enhances renal artery vascular compliance and reduces renal artery myogenic reactivity. Our meta-analysis shows that: 1. Pregnancy changes RVR, renal flow and GFR dependent on NO-activation and sympathetic de-activation, but adjustments are different among species, strains and gestational ages; 2. These changes do not depend on adaptation of renal artery responsiveness; 3. It remains unknown at which cellular level pregnancy affects the pathways. Our meta-analysis suggests that renal changes during pregnancy in animals are qualitatively similar, even in comparison to humans, but quantitatively different.

## Introduction

Profound vasodilation takes place in early pregnancy, and results in a major reduction in systemic vascular resistance. Vasodilation seems to be maximal at mid-gestation and slowly decreases towards term pregnancy in both humans [1] and animals [2]. Several local vascular responses, affecting the endothelial cell (EC), smooth muscle cell (SMC) and extracellular matrix, are thought to be responsible for gestational vasodilation by: 1. Up regulation of the endothelium-dependent nitric oxide (NO) pathway [3], 2. Reduction of the responsiveness to vasoconstrictor stimuli [4], 3. Reduction in myogenic reactivity [5], and 4. Increase in arterial compliance [5]. It remains to be determined to what extent each factor contributes to gestational vasodilation at the various stages of pregnancy among different species and strains.

Many studies on the mechanisms of vasodilation in pregnancy have focused on the kidney and its renal arteries. The kidney is a prominent vascular region and major contributor to systemic vascular resistance in the non-pregnant and pregnant condition [6], [7]. Vessel tone has been studied by investigating several local vascular responses. These responses can be divided by the stimulus involved: 1. Mechanical stimuli (flow-mediated vasodilation, myogenic reactivity, arterial compliance), and 2. Pharmacological stimuli, which can be subdivided by: a. The agent used (including acetylcholine, norepinephrine, phenylephrine, etc.) or b. The mediating receptor complex involved [8]. Most studies have concentrated on agents that affect the G-protein coupled receptors. These can be divided into three common G-protein coupled receptor pathways: the vasodilator Gq_EC_-pathway which mediates NO-dependent vasodilation, the vasoconstrictor Gq_SMC_-pathway which induces a calcium rise in the SMC, and the vasodilator Gs_SMC_-pathway which induces potassium influx in the SMC [8]–[15].

Various species, strains and gestational age periods have been investigated and often the results have been extrapolated to pregnancy-induced renal adaptation in general and to responsible mechanisms in humans. One may question the validity of such extrapolations. A previous meta-analysis showed that in mesenteric arteries vascular adaptation to pregnancy is strongly dependent on species, strains and gestational ages [8]. This may also be the case for renal arteries.

To investigate which mechanisms are responsible for renal vascular changes in pregnancy and whether they are dependent on species, strains and gestational age periods, we performed a systematic review and meta-analysis on the functional vascular changes of the kidney during pregnancy. Questions to be answered included: 1. Which specific renal vasodilator pathways are activated in pregnancy and to what extent do they affect RVR, renal flow and GFR across species, strains and gestational ages, 2. Are the pregnancy-induced changes in RVR, renal flow and GFR dependent on renal artery adaptation and which specific vasodilator pathways affect the renal artery during pregnancy, and 3. At which cellular level does pregnancy affect the involved pathways?

## Materials and Methods

### Retrieving the literature

In an attempt to identify all original studies on renal vasoactive responses in animals and humans during first pregnancy, we searched the Pubmed (from 1948) and Embase (from 1980) databases until April 2012. We used a three phase search strategy. Tables 1 and 2 depict the search-strategy for Pubmed and Embase, respectively. No language restriction was used in the primary literature search; later in the process non-English studies were excluded. We also searched the reference lists of included studies to identify additional studies. The search strategy was developed in cooperation with an information specialist from the Medical Library of the Radboud University Nijmegen.

**Table 1:** Literature search-strategy for Pubmed.

Component	Description
Pregnancy	*“pregnancy” [MeSH Terms] OR “pregnancy” [tiab] OR “pregnancies” [tiab] OR “gestation” [tiab] OR “pregnant” [tiab] OR “maternal-fetal relations” [tiab]*
Renal circulation	*“renal artery” [MeSH] OR “renal artery” [tiab] OR “renal arteries” [tiab] OR “kidney artery” [tiab] OR “kidney arteries” [tiab] OR “renal blood vessel” [tiab] OR “renal blood vessels” [tiab] OR “kidney blood vessel” [tiab] OR “kidney blood vessels” [tiab] OR “renal vessel” [tiab] OR “renal vessels” [tiab] OR “kidney vessel” [tiab] OR “kidney vessels” [tiab] OR “arteria renalis” [tiab] OR “arteria renis” [tiab] OR “kidney/blood supply” [MeSH] OR “renal circulation” [MeSH] OR “renal circulation” [tiab] OR “kidney circulation” [tiab] OR “renal blood circulation” [tiab] OR “kidney blood circulation” [tiab] OR “renal blood supply” [tiab] OR “kidney blood supply” [tiab]*
Vasoconstrictor and vasodilator responses	*“vasoconstriction” [MeSH Terms] OR “vasoconstriction” [tiab] OR “vasoconstrictions” [tiab] OR “vasoconstrictor agents” [MeSH Terms] OR “vasoconstrictor agents” [Pharmacological Action] OR “vascular resistance” [MeSH Terms] OR “vascular resistance” [tiab] OR “vascular capacitance” [MeSH Terms] OR (“vascular” [tiab] AND “capacitance” [tiab]) OR “vasoconstrictor” [tiab] OR “vasoconstrictors” [tiab] OR “vasopressor” [tiab] OR “vasoactive agonist” [tiab] OR “vasoactive agonists” [tiab] OR “vasopressors” [tiab] OR “vasomotor system” [MeSH Terms] OR “vasomotor system” [tiab] OR “peripheral resistance” [tiab] OR “artery constriction” [tiab] OR “vessel constriction” [tiab] OR “vasoconstrictive” [tiab] OR “vasoconstricting” [tiab] OR “vasoconstricted” [tiab] OR “vasodilation” [MeSH Terms] OR “vasodilation” [tiab] OR “vasodilatation” [tiab] OR “vasodilatating” [tiab] OR “vasodilating” [tiab] OR “vasodilative” [tiab] OR “vasodilatative” [tiab] OR “artery dilation” [tiab] OR “vessel dilation” [tiab] OR “artery dilatation” [tiab] OR “vessel dilatation” [tiab] OR “vasodilator agents” [MeSH Terms] OR “vasodilator agents” [Pharmacological Action] OR “vasodilator” [tiab] OR “vasodilators” [tiab] OR “vasorelaxation” [tiab] OR “Vascular Endothelium Dependent Relaxation” [tiab] OR “Endothelium Dependent Relaxation” [tiab] OR “Vascular Endothelium-Dependent Relaxation” [tiab] OR “Endothelium-Dependent-Relaxation” [tiab] OR “hemodynamics” [MeSH Terms] OR “hemodynamics” [tiab]OR “hemodynamic” [tiab] OR “vasodilated” [tiab] OR “vasoactive agent” [tiab] OR “vasoactive drug” [tiab] OR “vasoactive drugs” [tiab] OR “dilation” [tiab] OR “dilatation” [tiab] OR “contraction” [tiab] OR “relaxation” [tiab]*

**Table 2:** Literature search-strategy for Embase.

Component	Description
Pregnancy	*exp pregnancy/OR (pregnancy or pregnant).ti,ab. OR (pregnacies or gestation).ti,ab. OR (pregnancy or pregnant or pregnacies or gestation).ti,ab.*
Renal circulation	*exp renal artery/OR renal artery.ti,ab. OR renal arteries.ti,ab. OR arteria renalis.ti,ab. OR exp kidney artery/OR kidney artery.ti,ab. OR kidney arteries.ti,ab. OR arteria renis.ti,ab. OR kidney blood vessel/OR renal blood vessel.ti,ab. OR renal blood vessels.ti,ab. OR kidney blood vessel.ti,ab. OR kidney blood vessels.ti,ab. OR intrarenal vessel.ti,ab. OR intrarenal vessels.ti,ab. OR renal vessel.ti,ab. OR renal vessels.ti,ab. OR kidney vessel.ti,ab. OR kidney vessels.ti,ab. OR exp kidney blood flow/OR kidney blood flow.ti,ab. OR intrarenal blood flow.ti,ab. OR kidney bloodflow.ti,ab. OR renal blood flow.ti,ab. OR kidney blood supply.ti,ab. OR renal blood supply.ti,ab. OR exp kidney circulation/OR kidney circulation.ti,ab. OR intrarenal circulation.ti,ab. OR kidney blood circulation.ti,ab. OR renal circulation.ti,ab. OR renal blood circulation.ti,ab.*
Vasoconstrictor and vasodilator responses	exp vasoconstriction/OR exp vasoconstrictor agent/OR vasoconstrict*.ti,ab. OR exp vascular resistance/OR blood flow resistance.ti,ab. OR blood vessel resistance.ti,ab. OR (peripheral adj1 resistance).ti,ab. OR vascular vessel resistance.ti,ab. OR artery resistance.ti,ab. OR exp hemodynamics/OR exp blood vessel capacitance/OR blood vessel capacitance.ti,ab. OR vascular capacitance.ti,ab. OR (hemodynamic or hemodynamics).ti,ab. OR (haemodynamic or haemodynamics).ti,ab. OR vasopressor.ti,ab. OR exp vasoactive agent/OR (vasoactive agonist OR vasoactive agonists or vasoactive agent or vasoactive agents or vasoactive drug or vasoactive drugs).ti,ab. OR (vessel contriction or vessel contrictions or artery contriction or artery constrictions).ti,ab. OR exp vasodilatation/OR (vasodilatation or vasodilation or vasodilator or vasodilatator or vasodilating or vasodilative or vasodilatative).ti,ab. OR (vaso dilatation or vaso dilation or vaso dilator or vaso dilatator or vaso dilating or vaso dilative or vaso dilatative).ti,ab. OR vaso relaxation.ti,ab. OR vascular endothelium dependent relaxation.ti,ab. OR vascular endothelium-dependent relaxation.ti,ab. OR endothelium dependent relaxation.ti,ab. OR endothelium-dependent relaxation.ti,ab. OR (vessel dilatation* or vessel dilation* or vascular dilation* or vascular dilatation* or vasorelaxation* or artery dilatation* or artery dilation*).ti,ab. OR (dilatation* or dilation* or relaxation* or contraction*).ti,ab.

### Selection of studies and data extraction

The selection and data extraction process was divided into three phases. In the first phase, a. one investigator (first author) selected studies on the basis of title and abstract, and b. two investigators (first and second author) independently screened all remaining abstracts for inclusion criteria. Differences were resolved by mutual agreement. Studies were selected if they met the following criteria: 1. renal vasoconstrictor/vasodilator responses, 2. first pregnancy versus nulliparous non-pregnant control, 3. original data, 4.) healthy animals or humans, 5. age- or weight-matched subjects, and 6. for ex-vivo experiments: experiments performed in standard medium (defined as a generally accepted medium for the type of experiments). In the second phase of selection, non-English papers were excluded from further analysis and full articles were analyzed by two investigators (first and second author) independently using the same inclusion criteria as described above. Differences were resolved by mutual agreement. In the third phase, the first author extracted baseline measurements and responses to specific blockade from *in vivo* and whole organ perfusion experiments that related to renal function changes and the involved pathways. In addition, all vasodilator and vasoconstrictor responses were extracted both in the absence and presence of blockade of NO, prostaglandin (PGI_2_), renin-angiotensin-aldosterone (RAAS), endothelin, vasopressin or the sympathetic system, or combinations of blockade, with and without denuded endothelium. Responses had to contain ≥5 measurements to be included in further analysis, as analysis of sensitivity to stimuli is not accurate with fewer data points.

From each article, study data were extracted and recorded for species, strain, and gestational age (early, mid and late pregnancy, defined as first, second and third trimester, respectively), estrous/cycle stage, and experimental setup (*in vivo*/whole organ perfusion, myograph type used). We extracted all reported vascular responses and recorded for each of them the number of subjects, effect size, and standard deviation or standard error of the mean. For *in vivo* and whole organ perfusion experiments we detected baseline renal vascular resistance (RVR), renal flow (combined renal plasma, blood and perfusion flow), glomerular filtration rate (GFR), autoregulatory threshold (lower pressure limit for stable renal flow), and the response to specific blockades. For pharmacological responses, we extracted EC_50_ (concentration inducing 50% of the maximum effect) and E_max_ (maximum response to stimulus) to indicate sensitivity and maximum reactivity, respectively. For the mechanical responses, we determined the direction of the effect (favoring vasodilation, vasoconstriction, or no effect) and used it for descriptive analysis. Parametric comparison of these responses was not possible, as analyses (mostly ANOVA statistics) did not allow calculating overall effect sizes and effect measures were reported in variable ways (for myogenic reactivity and arterial compliance (absolute values, percentages of diameter changes, myogenic tone in relation to passive tone)). In case of missing, incomplete or indeterminate data, we approached the authors by email (response rate 33%).

### Quality assessment

The methodological quality of the articles was assessed independently by two reviewers (first and second author). Data were scored on presence or absence of randomization of allocation to groups, blinding of outcome assessment, clearness on nulliparity at entry of study, age or weight of subjects described, and number of animals accounted for at the start and the end of the study. The quality of the responses reported was ranked for the clarity of the number of animals used for analyses, presence of a response graph containing ≥5 measurements, and achievement of E_max_ (defined as the presence of at least two measurements without any further increasing effect). The response quality was expressed as percentage of the number of responses that complied with these items divided by the total number of responses described in the study. It should be noted that the quality assessment determines the quality of the methodology required for comparison of responses to the predefined items and that it should not be interpreted as judgment of the value of the experiment per se.

### Quantitative data synthesis and statistical analysis

Extracted data in RVR, renal flow and GFR at baseline, changes induced by specific blockades, and EC_50_ and E_max_ for pharmacological stimuli were analyzed for each gestational age period, stratified by species and strain, and displayed in forest plots, using Review Manager 5 (The Nordic Cochrane Centre, The Cochrane Collaboration, 2008). Pharmacological stimuli were analyzed by the type of G-protein-coupled pathway (Gq_EC_,Gq_SMC_ and Gs_SMC_) involved or by type of stimulus (NO-donors and potassium), when applicable. Combined effect sizes were calculated as weighed mean difference (MD) for RVR, renal flow, GFR and EC_50_, and as standardized mean difference (SMD) for E_max_. Both MD and SMD represent the effect sizes in comparison to control values within the same study. In the forest plots data were presented as mean effect sizes with 95% confidence intervals (CI), as provided by Review Manager 5. Combined effect sizes were calculated by using the random effects model (Review Manager 5). In the forest plots, the size of the boxes represents the weight of the studies, the gray diamonds depict the overall effect size per species and strain, and the black diamonds illustrate the overall effect sizes. We used a significance level of alpha  = 0.05 to identify statistically significant results. In the random effects model some heterogeneity beyond sampling errors is allowed to account for anticipated heterogeneity. Heterogeneity was presented as I^2^; I^2^<60% represents moderate heterogeneity, I^2^>60% represents considerable to substantial heterogeneity [16]. Publication bias was assessed by linear regression analysis as proposed by Egger et al. (for ≥10 studies) [17] or subjective assessment of funnel plot asymmetry (for ≤10 studies).

For mechanical stimuli, we used the direction of the measured effects for qualitative analysis, and determined the overall weighed direction for each mechanical response, based on the total number of animals used.

We performed sensitivity analysis to assess the robustness of our findings, as is recommended for systematic reviews [16]. This type of analysis shows the influence of the used inclusion and exclusion criteria on the results. In the analysis we repeated all analyses in the presence of the studies that were excluded on the basis of the criterion first pregnancy versus nulliparous non-pregnant control.

References used for analysis included the following: [18]–[54].

## Results

**Figure 1:**
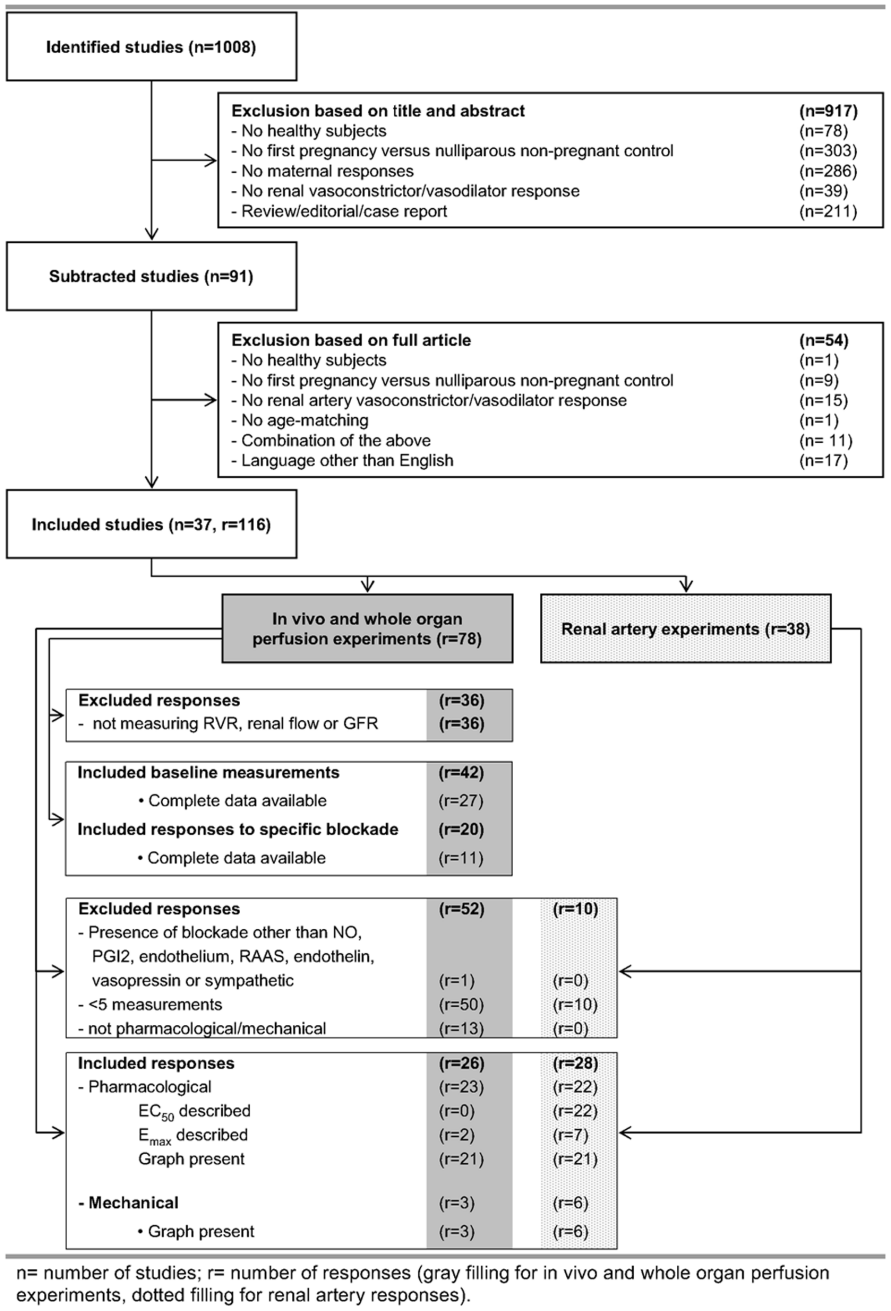
Flow chart for selection, inclusion and exclusion of studies and responses on renal vascular adaptation to pregnancy. n =  number of studies; r =  number of responses (gray filling for in vivo and whole organ perfusion experiments, dotted filling for renal artery responses).

Our systematic literature search identified 1008 studies concerning the effects of pregnancy on vascular changes of the kidney (Figure 1). Based on title and abstract 917 studies were excluded, leaving 91 papers for full article evaluation. Thirty-seven studies did not meet the inclusion criteria. Seventeen studies were excluded based on their non-English language. Finally, 37 studies with 116 vascular responses met the inclusion criteria for meta-analysis.

The study characteristics are presented in Table 3. Most studies (n = 28) used rats (Long Evans rats (LER), Sprague Dawley rats (SDR), various strains of Wistar rats (Wistar Kyoto (WKR), Munich Wistar (MWR) and Wistar Hannover (WHR)); other species were used less frequently (sheep  = 6, rabbits  = 2 and guinea pigs (GP)  = 1). Most studies contained *in vivo* experiments (n = 25), while whole organ (perfusion), perfusion myograph and wire myograph experiments were less common (n = 5, 3 and 4 respectively). Methodological limitations were present in a substantial portion of the included studies (Table 4): 43% (16/37) did not present the number of animals used, 32% (12/37) did not present parity, 97% (36/37) did not report having performed randomization, and 0% reported blinding of the outcome assessment.

**Table 3:** Characteristics of included studies, arranged by author and year.

Study	Species (strain)	Estrous stage control group	Gestation period	Experiment setup	Number of described baseline measurements	Number of described responses
Annibale et al., 1989	sheep	-	late	WM	n.a.	4
Baylis et al., 1986	rat (MWR)	-	late	IV	4	10
Baylis et al., 1988	rat (MWR)	-	mid	IV	3	0
Baylis et al., 1993	rat (SDR and MWR)	-	mid/late	IV	4	4
Baylis et al., 1995	rat (SDR)#	-	mid/late	IV	2	2
Bobadilla et al., 2001	rat (WR)	-	?	WOP	1	4
Bobadilla et al., 2003	rat (WR)	-	mid/late	WOP	2	16
Bobadilla et al., 2005	rat (WR)	-	late	WOP	1	2
Cha et al., 1993	sheep	-	late	IV	1	0
Cha et al., 1993	sheep	-	late	IV	1	0
Chu et al., 1997	rat (WKY)	-	late	WOP	0	0
Conrad et al., 1989	rat (LE)	-	late	IV	1	1
Conrad et al., 1999	rat (LE)	-	mid	IV	1	2
Danielson et al., 1995	rat (LE)	-	mid	IV	1	3
Danielson et al., 1996	rat (LE)	-	mid	IV	1	2
Fan et al., 1996	sheep	-	late	IV	1	0
Ferris et al., 1983	rabbit	-	late	IV	2	0
Gandley et al., 1997	rat (SDR)	-	late	PM	n.a.	4
Gandley et al., 2001	rat (LE)	-	mid	PM	n.a.	9
Greenberg et al., 1999	sheep	-	late	IV	1	0
Griggs et al., 1993	rat (SDR)	-	late	PM	n.a.	6
Hines et al., 1992	rat (SDR)	-	mid/late	IV	2	4
Hines et al., 2000	rat (SDR)	-	late	IV	1	1
Kim et al., 1994	guinea pig	-	Late	WM	n.a.	12
Knight et al., 2007	rat (SDR)	-	late	IV	0	0
Masilamani et al., 1994	rat (SDR)	-	mid/late	IV	1	0
McElvy et al., 2001	sheep	-	late	IV	1	0
Novak et al., 1997	rat (SDR)	-	mid	IV	1	0
Omer et al., 1999	rat (SDR)	-	late	IV	1	1
Patel et al., 1993	rat (SDR)	-	late	IV	2	0
Reckelhoff et al., 1992	rat (WR)	-	mid	IV	2	2
Sen et al., 1997	rat (?)	-	mid/late	IV	2	0
Sicinska et al., 1971	rat (SDR)	-	late	IV	1	0
Van Drongelen et al., 2011	rat (WR)	-	mid	WOP	1	1
Van Eijndhoven et al., 2003	rat (WR)	-	mid	WM	n.a.	1
Van Eijndhoven et al., 2007	rat (?)	-	mid	WM	n.a.	2
Woods et al., 1987	rabbit	-	late	IV	1	1

WM  =  wire myograph, PM  =  pressure myograph, PPM  =  pressure-perfusion myograph, WOP  =  whole organ perfusion, IV  =  *in vivo*. n.a.  =  not applicable.

# data received by email.

**Table 4:** Quality assessment of the included studies and subsequent responses.

	Study quality						Response quality			
Study	(1)	(2)	(3)	(4)	(5)	Score	(6)	(7)	(8)	Score
Annibale et al., 1989	-	-	?	-	+	1	1.0	1.0	1.0	3.0
Baylis et al., 1986	-	-	+	+	+	3	1.0	0.0	0.0	1.0
Baylis et al., 1988	-	-	+	+	-	2	1.0	0.0	0.0	1.0
Baylis et al., 1993	-	-	+	-	+	2	1.0#	0.0	0.0	1.0#
Baylis et al., 1995	-	-	+	+	-	2	1.0	0.0	0.0	1.0
Bobadilla et al., 2001	-	-	+	+	-	2	1.0	1.0	0.0	2.0
Bobadilla et al., 2003	-	-	+	+	+	3	1.0	1.0	0.0	2.0
Bobadilla et al., 2005	-	-	+	+	-	2	1.0	1.0	0.5	2.5
Cha et al., 1993^a^	-	-	?	+	+	2	0.0	0.0	0.0	0.0
Cha et al., 1993^b^	-	-	?	-	+	1	0.0	1.0	1.0	2.0
Chu et al., 1997	-	-	?	+	-	1	1.0	0.0	0.0	1.0
Conrad et al., 1989	n.a.	-	+	+	+	3	1.0	0.0	0.0	1.0
Conrad et al., 1999	-	-	+	+	-	2	0.0	0.0	0.0	0.0
Danielson et al., 1995	-	-	+	+	+	3	0.0	0.0	0.0	0.0
Danielson et al., 1996	+	-	+	+	-	3	1.0	0.0	0.0	1.0
Fan et al., 1996	-	-	?	-	+	1	1.0	0.0	0.0	1.0
Ferris et al., 1983	-	-	?	-	+	1	1.0	0.0	0.0	1.0
Gandley et al., 1997	-	-	+	+	-	2	1.0	1.0	0.75	2.75
Gandley et al., 2001	-	-	+	-	-	1	0.9	0.2	0.2	1.3
Greenberg et al., 1999	-	-	?	+	+	2	0.0	0.0	0.0	0.0
Griggs et al., 1993	-	-	+	+	-	2	1.0	0.16	0.33	1.49
Hines et al., 1992	-	-	+	-	-	1	1.0	0.0	0.0	1.0
Hines et al., 2000	-	-	+	-	+	2	0.33	0.66	0.0	1.0
Kim et al., 1994	-	-	?	-	-	0	1.0	1.0	1.0	3.0
Knight et al., 2007	-	-	+	+	+	3	1.0	0.0	0.0	1.0
Masilamani et al., 1994	-	-	+	+	+	3	1.0	0.0	0.0	1.0
Masilamani et al., 1994	-	-	+	+	+	3	1.0	0.0	0.0	1.0
McElvy et al., 2001	-	-	?	+	+	2	1.0	1.0	0.0	2.0
Novak et al., 1997	-	-	?	-	-	0	0.0	0.0	0.0	0.0
Omer et al., 1999	-	-	+	+	-	2	0.0	0.0	0.0	0.0
Patel et al., 1993	-	-	+	-	+	2	1.0	0.0	0.0	1.0
Reckelhoff et al., 1992	-	-	+	+	-	2	1.0	1.0	1.0	3.0
Sen et al., 1997	-	-	?	-	-	0	1.0	0.0	0.0	1.0
Sicinska et al., 1971	-	-	?	+	+	2	0.0	0.0	0.0	0.0
Van Drongelen et al., 2011	-	-	+	+	+	3	1.0	0.0	1.0	2.0
Van Eijndhoven et al., 2003	-	-	+	+	+	3	0.0	0.0	0.0	0.0
Van Eijndhoven et al., 2007	-	-	+	+	+	3	0.5	0.0	0.0	0.5
Woods et al., 1987	-	-	+	-	+	2	1.0	1.0	1.0	3.0

(1) randomization, (2) blinding of outcome assessor, (3) virgin/nulliparous at entry study, (4) age or weight of animals described, (5) number of used animals clear from methods, (6) fraction of responses with clear number of animals used for statistical analyses (as a percentage of the number of described responses), (7) fraction of responses with dose-response curve containing ≥5 measurements, (8) fraction of responses with accomplished E_max_. n.a.  =  not applicable.

# data received by email.

The 116 subtracted vascular responses could be divided into two categories: 1. *in vivo* and whole organ perfusion studies measuring changes in RVR, renal flow and/or GFR, and 2. renal artery experiments measuring changes in vascular tone. Experiments were performed mainly in mid (34%, 40/116) and late (63%, 73/116) gestation; for four responses the gestation period was not defined. Early gestation was not investigated by any of the included studies.

We identified 78 *in vivo* and whole organ perfusion experiments. Two types of experiments could be distinguished based on their objective to determine the effects of pregnancy on: 1) RVR, renal flow and GFR, or 2) the response of the kidney to vaso-active substances. For the first type of experiments we extracted 42 baseline measurements and 20 responses to specific blockades. Thirty-six responses were excluded as they did not measure RVR, renal flow or GFR. After exclusion of incomplete data, 27 baseline measurements and 11 responses to specific blockades were suitable for meta-analysis. For the second type of experiments we identified 23 pharmacological and 3 mechanical responses. For pharmacological stimuli, EC_50_ was reported in 0% of the responses, E_max_ was described in 9% and a graph was present in 91%. For mechanical stimuli, all responses were presented graphically without quantified effects.

From the 38 renal artery responses, 28 met the criteria for inclusion. They consisted of 22 pharmacological stimuli (EC_50_, E_max_ and a graph present in 100%, 32% and 95% of the cases, respectively) and six mechanical stimuli, all with a graph without quantified effects.

### *In vivo* and whole organ perfusion experiments

**Figure 2:**
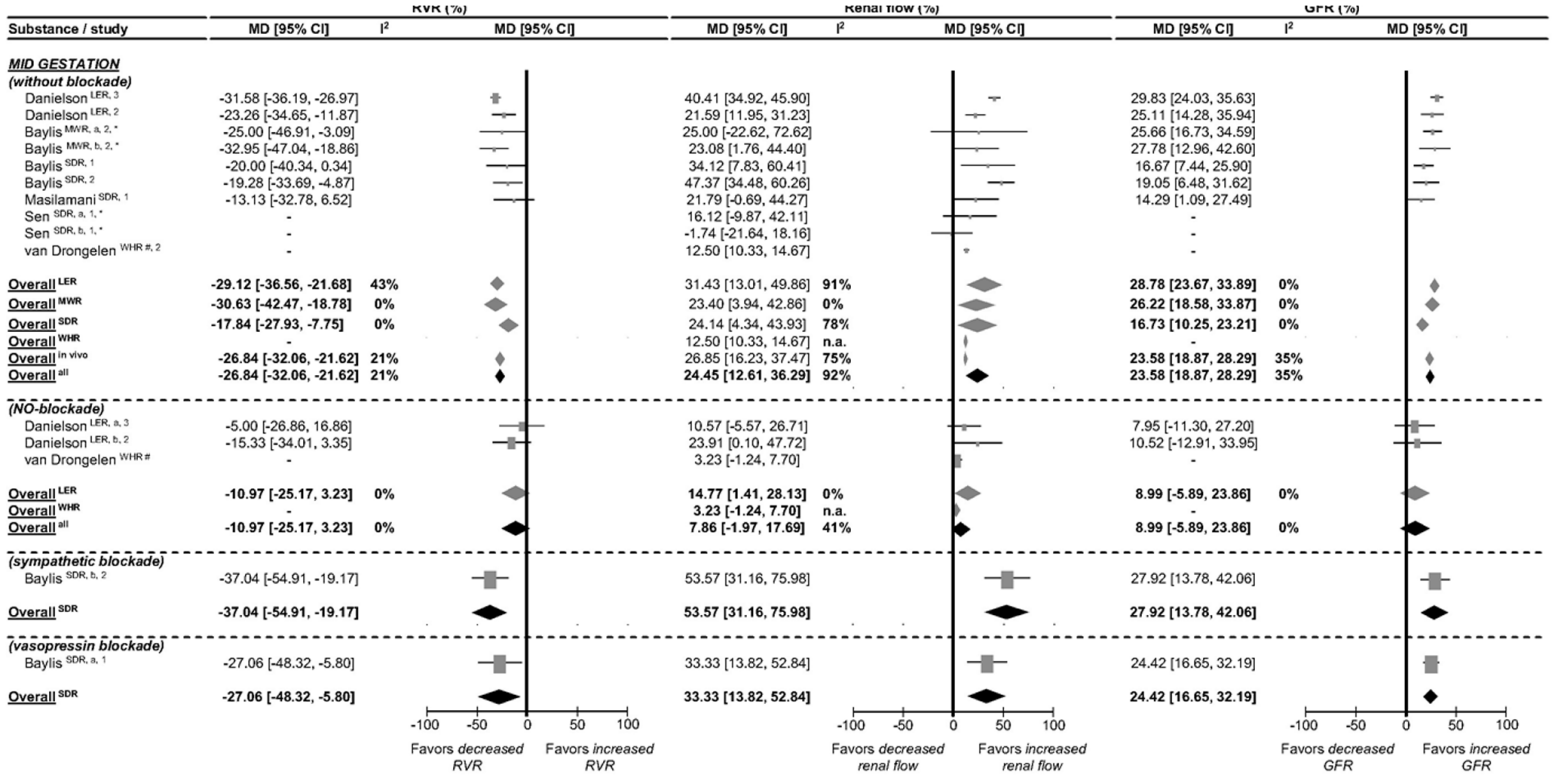
Effect of pregnancy at mid gestation on in vivo and whole organ perfusion# renal vascular resistance (RVR), renal flow and glomerular filtration rate (GFR) in absence and presence of NO and sympathetic blockade. The effect of pregnancy on RVR, renal flow (effects of renal plasma/blood/perfusion flow (RPF/RBF/RPPF) combined) and GFR is presented as percentage mean difference (MD) and its 95% CI. Studies and totals based on Long Evans rats (LER), Munich Wistar rats (MWR), Sprague Dawley rats (SDR) and Wistar Hannover rats (WHR). 1, 2, 3 represents first, second and third part of mid gestation. # Whole organ perfusion experiments, excluded in the “Overall in vivo“ analysis. * Experiments performed under anesthesia. I^2^ represents the amount of heterogeneity. n.a.  =  not applicable.

The influence of mid pregnancy on kidney function is presented in Figure 2. In mid pregnancy, *overall* (across species and strains) RVR decreases by 27%, varying from 18% in SDR to 31% in MWR. Renal flow is enhanced to a variable degree, ranging from 13% in WHR to 31% in LER. GFR increases by 17% in SDR to 29% in LER. In LER, blockade of the NO system completely normalizes the pregnancy-induced change in RVR and GFR, while pregnancy-induced increase in renal flow is reduced from 31% to 15%. In WR, NO-blockade normalizes renal flow completely. Sympathetic or vasopressin blockade has only been studied in SDR and does not affect the mid pregnancy-induced changes in RVR, renal flow and GFR. It appears that mid pregnancy changes in RVR, renal flow and GFR are dependent on up regulation of the NO system and not on sympathetic tone or vasopressin.

**Figure 3:**
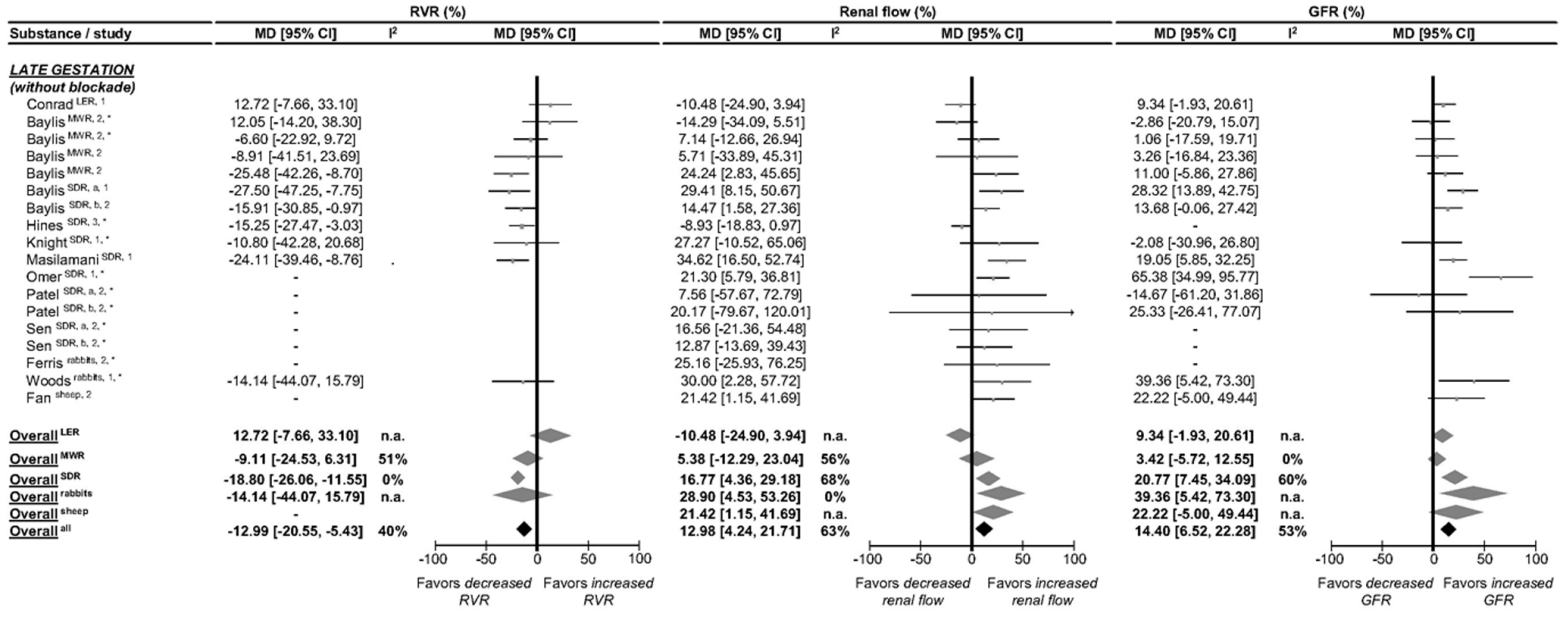
Effect of pregnancy at late gestation on in vivo renal vascular resistance (RVR), renal flow and glomerular filtration rate (GFR) in absence of blockade. The effect of pregnancy on RVR, renal flow (effects of renal plasma/blood/perfusion flow (RPF/RBF/RPPF) combined) and GFR is presented as percentage mean difference (MD) and its 95% CI. Studies and totals based on Long Evans rats (LER), Munich Wistar rats (MWR), Sprague Dawley rats (SDR), rabbits and sheep. 1, 2, 3 represents first, second and third part of late gestation. * Experiments performed under anesthesia. I^2^ represents the amount of heterogeneity. n.a.  =  not applicable.

**Figure 4:**
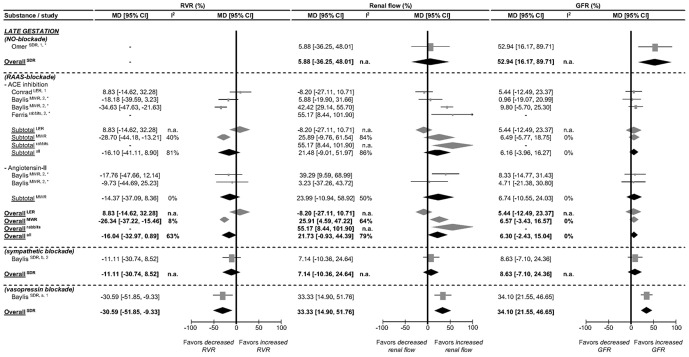
Effect of pregnancy at late gestation on in vivo renal vascular resistance (RVR), renal flow and glomerular filtration rate (GFR) in presence of NO, sympathetic and RAAS blockade. The effect of pregnancy on RVR, renal flow (effects of renal plasma/blood/perfusion flow (RPF/RBF/RPPF) combined) and GFR is presented as percentage mean difference (MD) and its 95% CI. Studies and totals based on Long Evans rats (LER), Munich Wistar rats (MWR), Sprague Dawley rats (SDR) and rabbits. 1, 2, 3 represents first, second and third part of late gestation. * Experiments performed under anesthesia. I^2^ represents the amount of heterogeneity. n.a.  =  not applicable.

The magnitude of pregnancy-induced renal changes in rats decreases from mid pregnancy towards term. Figures 3 and 4 show renal function in late gestation compared to non-pregnant controls. Late pregnancy affects RVR, renal flow and GFR differently among species and strains. In LER and MWR, pregnancy does not affect any of the renal parameters. Blockade of the renin-angiotensin-aldosteron system (RAAS) did not show any effect in LER, but in MWR reduced RVR by 26% and increased renal flow by 26% without changing in GFR. In SDR, pregnancy decreases RVR by 19% and increases both renal flow and GFR, by 17% and 21% respectively. NO blockade in this strain returns renal flow to non-pregnant values with a persistently increased GFR. Under sympathetic blockade RVR, renal flow and GFR return to non-pregnant values in these rats, while vasopressin blockade has no effect on any of the parameters. In rabbits, late pregnancy does not significantly affect RVR, while renal flow and GFR are increased by 29% and 39% compared to non-pregnant values. These changes are not affected by RAAS-blockade. For late pregnant sheep, no data are available on RVR, but renal flow is 21% higher than in non-pregnant sheep without a significant difference in GFR. In short, there is no consistent pattern of renal changes in late pregnancy across species and strains.

In both mid and late pregnancy, the *overall* RVR, renal flow and GFR shows moderate to considerable heterogeneity. This cannot be attributed completely to species or strain specific effects, as stratification by species and/or strain does not reduce heterogeneity to a minimum in all groups. Heterogeneity depends mainly on single responses with very small confidence intervals and responses with an estimated effect opposite to other studies. We could not identify any methodological issue that might explain these effects.

The influence of pregnancy on the autoregulatory threshold was analyzed qualitatively only, as quantitative effect measures of the lower pressure boundary for stable renal flow were not reported in the two relevant studies, which used WR and rabbits [21], [54]. From these studies it appears that pregnancy does not affect the renal autoregulatory threshold in either mid or late pregnancy.

### Renal artery experiments

**Figure 5:**
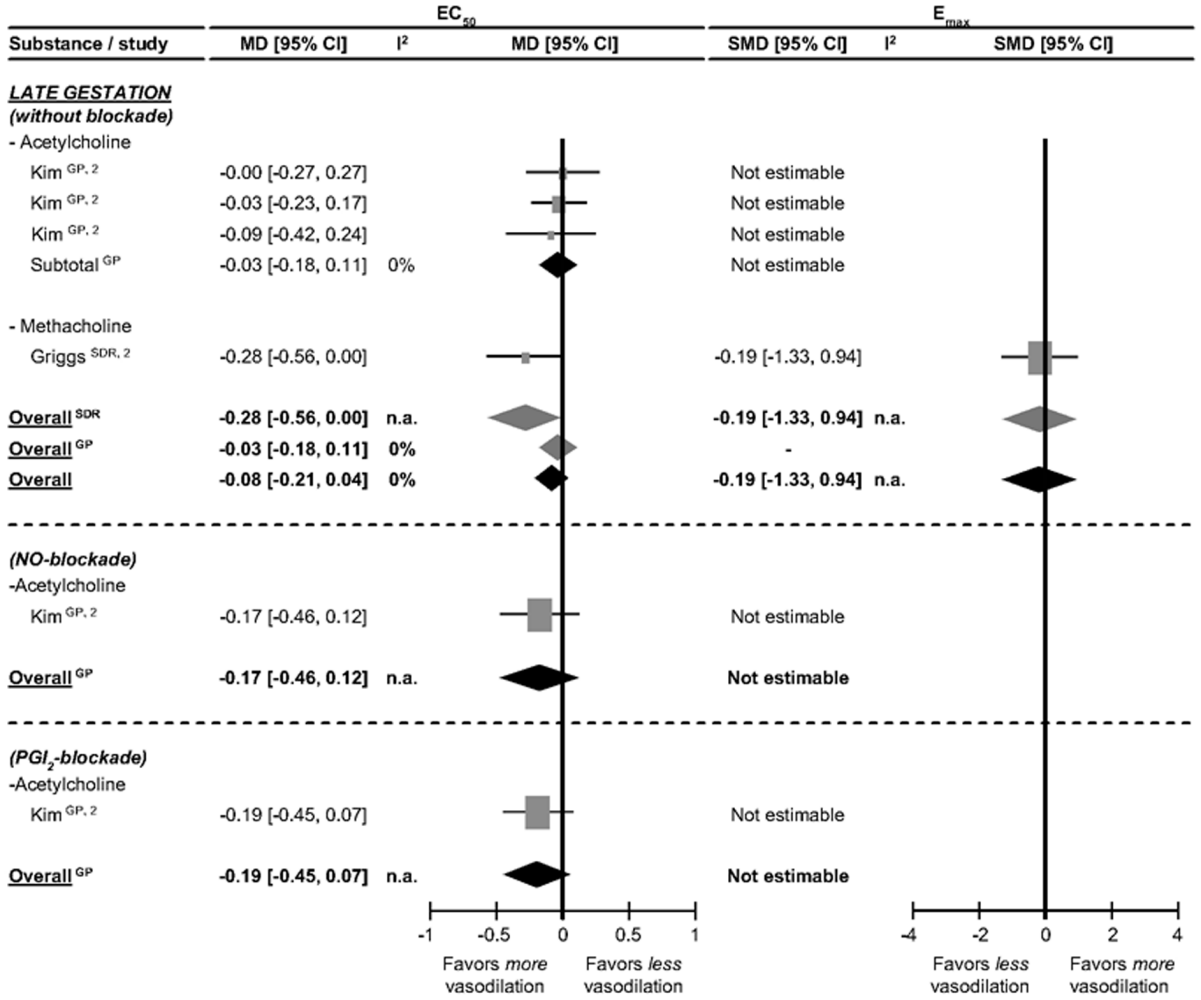
Effect of pregnancy at late gestation on renal artery responses to stimuli involved in vasodilation through the GqEC-coupled pathway in the presence and absence of blockade. The effect of pregnancy on EC_50_ (the dose of stimulus inducing 50% response) is depicted as mean difference (MD) and its 95% confidence interval (95% CI). E_max_ (maximum response) is presented as standardized mean difference (SMD) and its 95% CI. Studies and totals based on Sprague Dawley rats (SDR) and guinea pigs (GP). 1, 2, 3 represents first, second and third part of late gestation. I^2^ represents the amount of heterogeneity. n.a.  =  not applicable.

The renal artery changes in response to vasodilator Gq_EC_-coupled pharmacological stimuli in pregnancy are presented in Figure 5. Our search only detected responses during late gestation, and mainly in guinea pigs. In this species, pregnancy does not affect Gq_EC_-coupled EC_50_ to acetylcholine and EC_50_ is unaffected by NO and PGI_2_ blockade.

**Figure 6:**
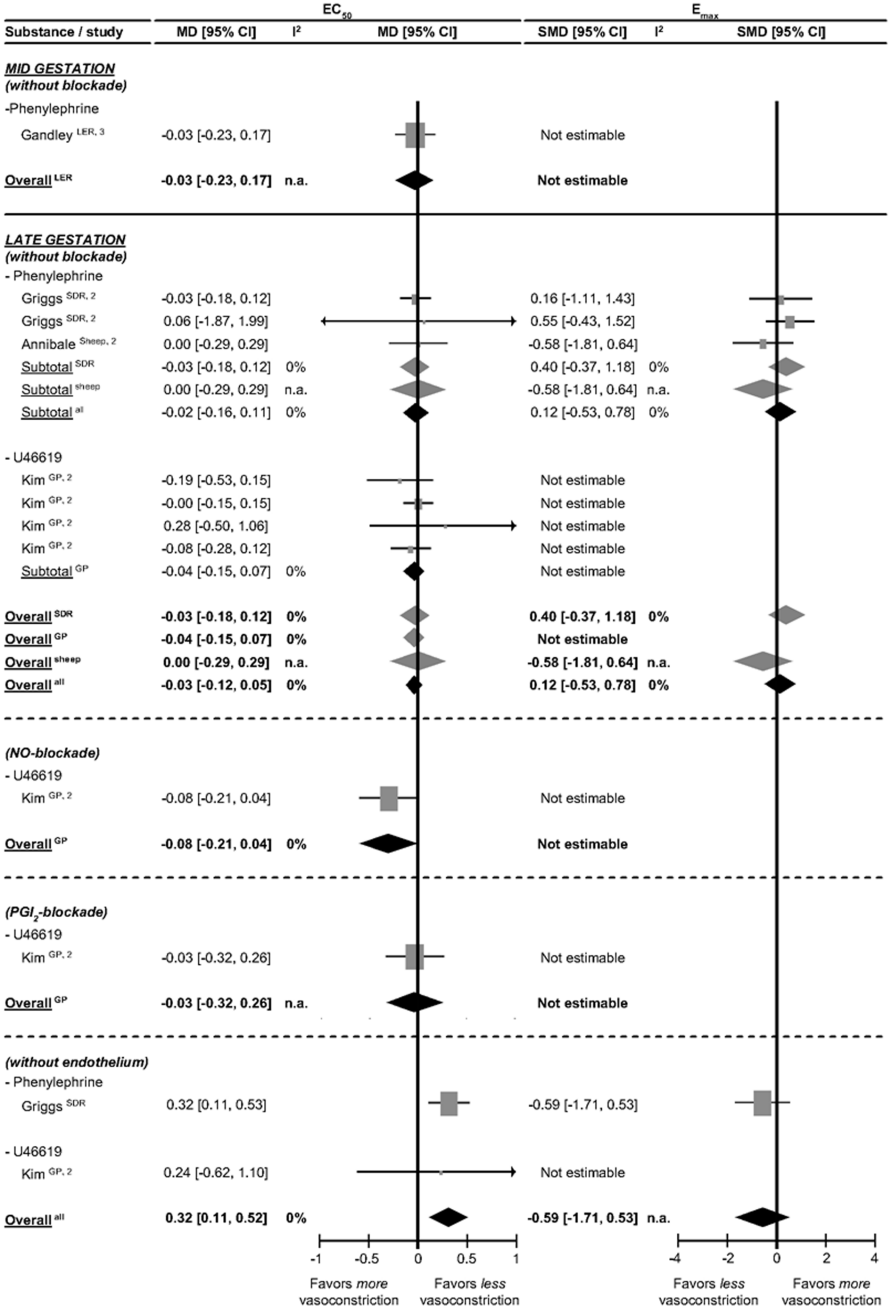
Effect of pregnancy at mid and late gestation on renal artery responses to stimuli involved in vasoconstriction through the GqSMC-coupled pathway in presence and absence of blockade. The effect of pregnancy on EC_50_ (the dose of stimulus inducing 50% response) is depicted as mean difference (MD) and its 95% confidence interval (95% CI). E_max_ (maximum response) is presented as standardized mean difference (SMD) and its 95% CI. Studies and totals based on Long Evans rats (LER), Sprague Dawley rats (SDR), sheep and guinea pigs (GP). 1, 2, 3 represents first, second and third part of mid and late gestation. I^2^ represents the amount of heterogeneity. n.a.  =  not applicable.

The effects of mid and late pregnancy on the vasoconstrictor Gq_SMC_-coupled pathway in several species are shown in Figure 6; no data are available on early gestation. In both mid and late gestation, the Gq_SMC_-coupled EC_50_ and E_max_ of phenylephrine and U46619 (thromboxane agonist) are unaffected by pregnancy for all species investigated. In guinea pigs under NO and PGI_2_ blockade, pregnancy does not affect the response to U46619. In SDR in the absence of endothelium pregnancy increases EC_50_ in response to phenylephrine, which suggests that the pregnancy effect on the SMC is overruled by the endothelium. Apparently, pregnancy does not seem to affect the renal artery responsiveness to Gq_SMC_-coupled vasoconstrictor stimuli.

**Figure 7:**
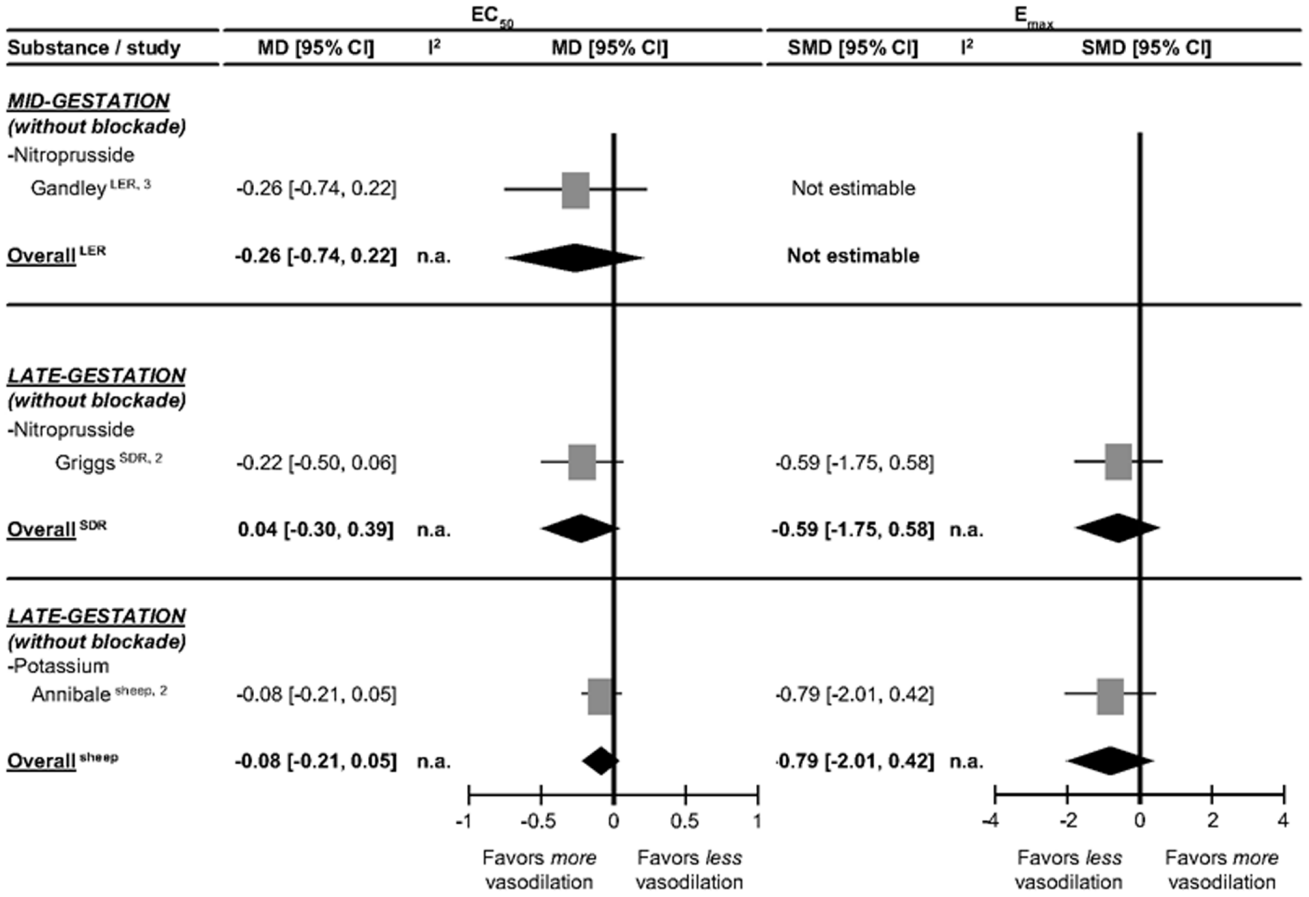
Effect of mid and late pregnancy on renal artery responses to nitric oxide (NO) and potassium. The effect of pregnancy on EC_50_ (the dose of stimulus inducing 50% response) is depicted as mean difference (MD) and its 95% confidence interval (95% CI). E_max_ (maximum response) is presented as standardized mean difference (SMD) and its 95% CI. Studies and totals based on Long Evans rats (LER), Sprague Dawley rats (SDR) and sheep. 1, 2, 3 represents first, second and third part of mid and late gestation. I^2^ represents the amount of heterogeneity. n.a.  =  not applicable.

The effect of pregnancy on the renal artery responses to NO and potassium are depicted in Figure 7. For NO, only single studies in rats and sheep were available. In mid and late gestation rats and late pregnant sheep pregnancy does not modify the response to NO or potassium. This suggests that in general pregnancy does not affect the properties of the renal SMC to NO and potassium.

**Figure 8:**
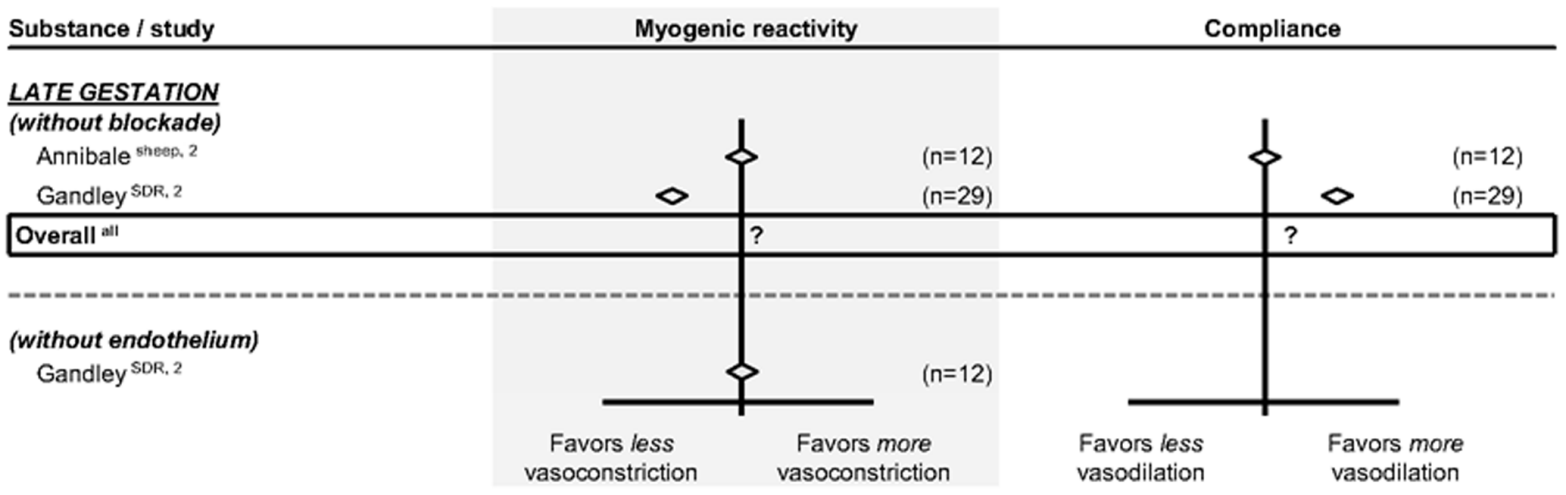
Effect of pregnancy at late gestation on renal artery responses to myogenic response and vascular compliance. The effect of pregnancy on the response to stimuli, depicted as the difference in direction.?  =  unknown or non-conclusive data. Studies and totals based on Sprague Dawley rats (SDR) and sheep. 1, 2, 3 represents first, second and third part of late gestation. n =  number of subjects.

The effect of pregnancy on the renal artery responses to mechanical stimuli was assessed qualitatively only, as shown in Figure 8. We did not find studies that investigated flow-mediated vasodilation of the renal artery during pregnancy, and only two studies that looked at myogenic reactivity and vascular compliance in late gestation. In SDR, late pregnancy reduces myogenic reactivity in an endothelium-dependent manner and enhances vascular compliance. In sheep, late pregnancy does not affect either myogenic reactivity or vascular compliance. Apparently, pregnancy-induced changes in myogenic reactivity and compliance depend on the species investigated.

### Publication bias and sensitivity-analysis

Publication bias was assessed by subjective determination of funnel plot asymmetry, as all analyses consisted of less than 10 studies. We did not detect evidence suggestive for publication bias.

Sensitivity-analysis was performed to assess the influence of inclusion criteria on the results. We extended the inclusion for studies that did not match the criterium “nulliparity” (n = 3). The results were not affected by addition of these studies (data not shown). We did not extend the analysis for age-matching, because we had not excluded any study for that reason.

## Discussion

Our meta-analysis confirms the commonly held view that pregnancy reduces RVR and enhances renal flow and GFR in animals, as it does in humans [1]. Our study has shown that the degree of change depends on the functional parameter, species, strain and gestational age investigated. Our meta-analysis did not detect changes in renal artery responsiveness. More importantly, the underlying mechanisms for the change in RVR, renal flow and GFR is not the same between species, strains and gestational age. Therefore each research question needs careful choice of the animal model and gestational age period of interest.

Across rat strains at mid-gestation, pregnancy decreases RVR and enhances renal flow and GFR, as shown in Figure 2. The effect of pregnancy on RVR and GFR is quite consistent, whereas there is considerable variation in renal flow. This may suggest differences in renal blood pressure between strains. We observed that the common changes in RVR, renal flow and GFR are caused by NO upregulation rather than by change in renal autoregulatory threshold to pressure, or sympathetic or vasopressin-regulation.

At late gestation, the pregnancy effects on RVR, renal flow and GFR are dependent on the species and strain investigated (Figure 3). In some species or strains, renal parameters are not different from non-pregnant values (LER and MWR), while in others late pregnancy affects RVR, renal flow and GFR (SDR, rabbits and sheep). The renal autoregulatory threshold to pressure changes was not affected in any species investigated. The NO, RAAS, sympathetic and vasopressin pathways are differently affected across species and strains. In SDR, NO activation affects renal flow, but not GFR. This implies a difference in response to NO activation between the afferent and efferent glomerular vasculature.

**Figure 9:**
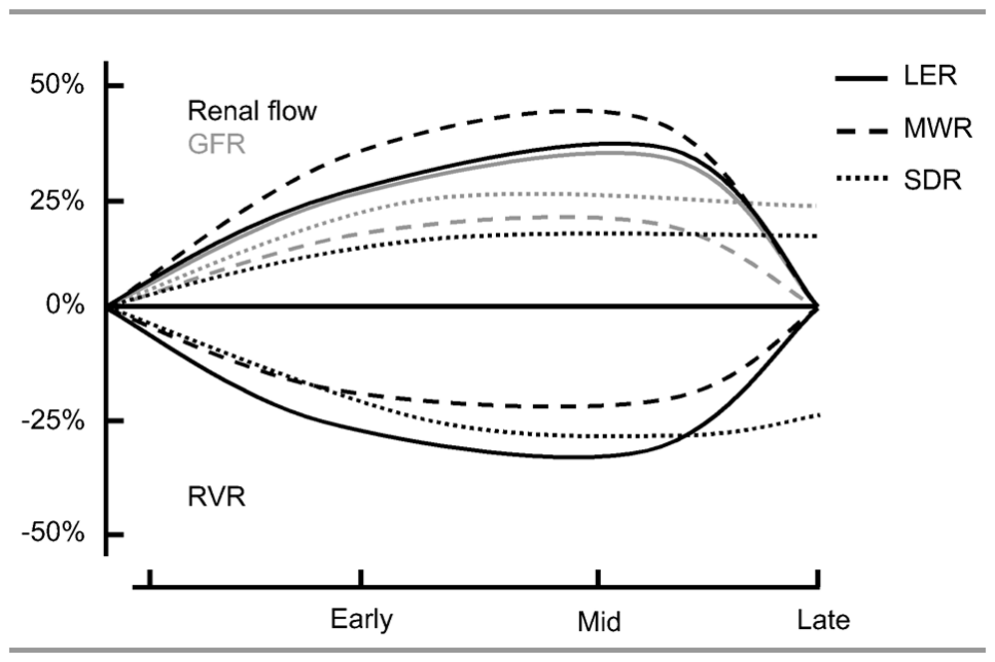
Summary of renal vascular resistance (RVR), renal flow and glomerular filtration rate (GFR) during pregnancy in Long Evens rats (LER), Munich Wistar rats (MWR) and Sprague Dawley rats (SDR). Graph design based on data at mid- and late gestation. No data were available on early pregnancy.

The pregnancy-induced reduction in RVR and the associated increase in renal flow and GFR across the species studied are dependent on gestational age, as shown in Figures 2 and 3. Our analysis includes data on both mid- and late gestation in LER, MWR and SDR (Figure 9). In LER and MWR, all measured renal changes are maximal in mid-gestation and return towards non-pregnant levels in late gestation, whereas in SDR there is no difference between mid- and late pregnancy response. Responses in LER and MWR qualitatively correspond well with human pregnancy, in which renal flow increases in the first and second trimesters and decreases markedly towards term [1]. Quantitatively, in human pregnancy the renal flow increase (around 60%) is more pronounced than in the rat strains included in our meta-analysis (LER 31%, MWR 45% and SDR 14%). One may speculate on the underlying mechanism for this difference. Possibly the more mature human fetus poses a greater demand on the maternal vascular system, than the more immature rat fetus at comparable gestational age period. Our data suggest that renal changes during pregnancy in humans and rats are qualitatively similar but quantitatively different.

It is questionable if SDR is the right model for renal changes in healthy human pregnancy. In contrast to humans in which RVR, renal flow and GFR return towards non-pregnant values near term, the changes in SDR are maximal in mid-pregnancy and remain at maximum level throughout late pregnancy. As shown in Figure 9, this pattern is different from that in the other rat strains investigated (LER, MWR), that matches better with the human pattern. The persistent changes in SDR may reflect their relatively compromised vascular health. This strain is known to develop severe vascular dysfunction later in life, including chronic progressive nephropathy, peri-/vasculitis, and chronic cardiomyopathy [55]. SDR could be the better model for renal vascular maladaptation, whereas LER and MWR seem the more appropriate models for normal pregnancy-induced renal vasodilation.

Our meta-analysis detected considerable heterogeneity (I^2^>60%) for overall renal flow and moderate heterogeneity (I^2^<60%) for overall GFR and RVR. This can only partially be attributed to differences between species and strains, as stratification reduced heterogeneity substantially only for some subgroups. Additionally, methodological differences in determining renal flow (flow probe or para-aminohippurate clearance) may have contributed to the heterogeneity. The substantial degree of heterogeneity implies that data should not be quantitatively extrapolated to other species or strains.

Renal artery function is not affected by pregnancy, except for SDR. Pregnancy does not change the renal artery responsiveness to pharmacological stimuli (Gq_EC_- and Gq_SMC_-mediated stimuli, NO and potassium). One study in SDR showed an endothelium-dependent decrease in renal artery myogenic reactivity and increased vascular compliance in late pregnancy. Apparently SDR activate additional mechanisms to realize the same degree of vascular adaptation to pregnancy, as compared to other strains. This may represent a more generalized pattern, as it has been reported that SDR activate additional vascular adaptive pathways also in mesenteric arteries responses in pregnancy [8].

One may question what mechanisms are responsible for the changes in RVR, renal flow and GFR in pregnancy, given the lack of a role for renal artery adaptation. Our meta-analysis does not provide the answer. Several mechanisms can be considered. Reduced RVR and enhanced renal flow and GFR in pregnancy most likely result from regulation by the small resistance vessels of the kidney, rather than the renal artery [56]. The juxtaglomerular apparatus, which regulates afferent and efferent glomerular artery tone, may also be involved through resetting of the tubuloglomerular feedback system [57]. It seems likely that pregnancy-specific hormones, including relaxin or progesterone, play a role in these processes [58], [59].

Several methodological aspects of our meta-analysis deserve discussion. First, the quality of all included studies was scored as poor, in terms of the reported number of animals, parity, randomization, and blinding the outcome assessment. This is a common finding in animal experiments [8], which are primarily concerned with generation and testing of hypotheses rather than with rigorous employment of randomized-controlled-trial methodology. Nonetheless, the quality of animal experimental work, and therefore the reliability of its findings, could benefit from application of strict methodological criteria, standardized procedures and reporting guidelines [60]. Second, our literature search was not designed to identify all studies that reported on pregnancy-induced changes in RVR, renal flow and GFR. Because we were primarily interested in the underlying mechanisms, we restricted our search to studies investigating responses to vaso-active stimuli. Many studies in different animals have investigated RVR, RPF and GFR without using vaso-active stimuli. These studies were not included in our meta-analysis. Our data on pregnancy-induced changes imply species, strain and gestational age differences in RVR, RPF and GFR, without having the intention to be complete on this. Third, despite extensive searches, some of our observations are still based on a limited number of animals and strains of animals. Obviously, these results have to be interpreted with some restraint. Fourth, publication bias may have affected the results. Negative results tend to be underreported, which may therefore lead to overestimated effect sizes. Our funnel-plot analysis did not detect any such effect. Fifth, one may question whether it is legitimate to group together responses to pharmacological stimuli across species and strains. We observed considerable heterogeneity (I^2^>60%), which implies that one should not regard the responses as uniform across species and strains. Sixth, one may question the validity of combining the responses to different stimuli according to their assumed common G-protein coupled pathway. If heterogeneity would have been low, it would have been reasonable to group together the various stimuli to their common pathways, as observed in a former study of our group [61]. The moderate heterogeneity, observed for most responses in the present study, might imply that the assumption is not valid. However, it does not necessarily disprove the validity, because heterogeneity may be affected by many methodological aspects of the experiments. Given the moderate heterogeneity, one should mainly focus on the qualitative similarities.

In conclusion, our meta-analysis shows that pregnancy reduces RVR and increases renal flow and GFR through NO activation and sympathetic de-activation and not through a change in renal artery responsiveness. The cellular level at which pregnancy affects the respective pathways remains unknown. Quantitatively, renal vascular changes in pregnancy vary between species, strains and gestational age. Our meta-analysis suggests that renal changes during pregnancy are qualitatively similar in animals and even in comparison to humans, but quantitatively different.

## Supporting Information

Checklist S1 Prisma Checklist.
(DOC)

Data S1 RevMan export data file.
(CSV)
